# Cleaner Leather Tanning and Post-Tanning Processes Using Oxidized Alginate as Biodegradable Tanning Agent and Nano-Hydroxyapatite as Potential Flame Retardant

**DOI:** 10.3390/polym15244676

**Published:** 2023-12-11

**Authors:** Ilaria Quaratesi, Maria Cristina Micu, Erica Rebba, Cristina Carsote, Noemi Proietti, Valeria Di Tullio, Rita Porcaro, Elena Badea

**Affiliations:** 1 National Research and Development Institute for Textile and Leather (INCDTP), Research Institute for Leather and Footwear Branch (ICPI), Ion Minulescu Str. 93, 031215 Bucharest, Romania; i.quaratesi@gmail.com (I.Q.); 711mariacristina@gmail.com (M.C.M.); 2 Department of Chemistry, NIS Interdepartmental and INSTM Reference Centre, University of Turin, Via Pietro Giuria 7, 10125 Torino, Italy; erica.rebba@unito.it; 3 National Museum of Romanian History, Calea Victoriei Str. 12, 030026 Bucharest, Romania; criscarsote@yahoo.com; 4 Istituto di Scienze del Patrimonio Culturale (ISPC), Consiglio Nazionale delle Ricerche (CNR), Area della Ricerca di Roma 1, 00015 Monterotondo, RM, Italy; noemi.proietti@cnr.it (N.P.); valeria.ditullio@cnr.it (V.D.T.); 5 KEMIA TAU SRL, Via Torino 56/64, 10040 La Cassa, TO, Italy; rita.porcaro@kemiatau.com; 6 Department of Chemistry, Faculty of Sciences, University of Craiova, Calea Bucuresti Str. 107 I, 200512 Craiova, Romania

**Keywords:** oxidized sodium alginate, nano-hydroxyapatite, tanning agent, flame retardant, clean technology, sustainable leather

## Abstract

In this study, sodium alginate (SA) was oxidized with potassium periodate to produce an alginate-based tanning agent. Using OSA as a biodegradable tanning agent and a nano-hydroxyapatite (nano-HAp) low concentration suspension to give flame retardancy to leather, eco-design concepts were applied to establish a chrome-, aldehyde-, and phenol-free tanning process. Micro-DSC, ^1^H unilateral nuclear magnetic resonance (NMR), attenuated total reflection mode Fourier transform infrared spectroscopy (FTIR-ATR), and scanning electron microscopy with energy dispersive X-ray spectroscopy (SEM-EDS) were used to investigate the complex matrix collagen-OSA-nano-HAp. Micro-differential scanning calorimetry (micro-DSC) was used to assess OSA’s ability to interact with collagen and stabilize the collagen-OSA matrix, while ^1^H unilateral (NMR) was used to investigate the aqueous environment and its limitations around collagen molecules caused by their association with OSA and nano-HAp. Industrial standard tests were used to assess the mechanical properties and fire resistance of the new leather prototype. The findings reported here indicate that both OSA and nano-HAp are suitable alternatives for cleaner tanning technologies and more sustainable leather.

## 1. Introduction

### 1.1. Sustainability Issues in the Tanning Industry

In the last hundred years, chrome tanning has been the dominant method of making leather due to both the economic advantages and ease of achieving versatile end products with high performances for many traditional and modern applications. Leather, the first biomaterial made by man, is still irreplaceable due to its properties unmatched by other synthetic materials. However, chrome tanning went into re-evaluation for its hazardous wastes contaminating water, soil, and air, especially through the possible oxidation of Cr^3+^ to Cr^6+^, classified as a human carcinogen [1,2,3]. Besides, around 4% chromium is found in the finished products [4,5] making leather practically non-biodegradable and difficult to be reused. Worldwide, huge amounts of the chromium tanned leather wastes are discarded to landfills by the leather industries annually worldwide [6,7]. This means wasting of the contained proteinaceous resources while being a cause of environmental concern. On the other hand, wet white tanning solution proved equally harmful, with aldehyde-based tannins demonstrating effects such as carcinogenic and teratogenic properties and the release of formaldehyde from commercial tanning agents. For example, glutaraldehyde- and oxazoline-based tanning agents cannot meet the formaldehyde limit, as these tanning agents continuously decompose and release formaldehyde during the production, storage, and use of leather [8]. In addition, bisphenol S-based polymers shown to have endocrine and estrogenic activity [9,10].

The tanning operation is the main contributor to the environmental impacts of a tanning system followed by re-tanning and fatliquoring operations. Thus, more sustainable tanning agents than chrome salts, bisphenol-based tannins, and synthetic aldehydes have become the objective of much research in recent decades [7,11].

Directive 2005/64/EC of the European Parliament on the re-use, recycling and recovery of vehicle parts and materials, as well as the continuously tightening regulatory framework for safer and cleaner chemicals and technologies (i.e., REACH, the regulation of the European Union, aimed to improve the protection of human health and the environment from the risks that can be posed by chemicals), pushes towards developing innovative metal-, aldehyde-, and phenol-free tanning technologies. The increased customers demand safe, durable, and functional leather goods manufactured in a sustainable manner has created an opportunity for alternative tanning technologies based on safe and non-toxic bio renewable resources and nanomaterials as active compounds to play the role of new future auxiliaries for hide and leather treatments. Sustainable tanning technologies aiming to produce 100% biodegradable leather are highly sought to keep the image of leather as synonymous with quality, both aesthetic and functional, and sustainability in compliance with the concept of ethical and moral purchase, which tends to replace the consumerism of previous decades.

In this context, we set out to develop and test tanning agents based on sodium alginate and test the potential ability of nano-HAp to impart flame-retardant properties on leather with the aim of taking essential steps to obtain a biodegradable leather with superior mechanical, thermal and water resistance qualities.

### 1.2. Oxidized Sodium Alginate as an Alternative Chrome-Free Tanning Material

The analysis of the literature revealed that the development of alginate oxidation approaches has expanded the potential of alginate applications, including the tanning industry as well [12,13,14,15,16]. Alginates are considered one of the world’s most abundant polysaccharides, accounting for up to 40% of the dry matter of seaweed. At the same time, their industrial production is inexpensive. Alginates are anionic linear copolymers consisting of block copolymers comprising 1,4-linked β-D-mannuronic acid (M) with 4C1 ring conformation and α-L-guluronic acid (G) with 4C1 conformation, both in the pyranosic conformation and present in varying proportions [17]. Due to their non-toxicity, non-carcinogenicity, and biocompatibility with the human body, applications of alginates (in the form of alginic acid and its sodium or calcium salts) in food, cosmetics, medicine, and tissue engineering have been extensively studied [18]. Sodium alginate (SA) undergoes partial oxidation by NaIO_4_/KIO_4_ and loses molecular weight while gaining more reactivity-high aldehyde groups. Two of these groups form in each oxidized uronic acid subunit [19,20,21]. The resulting oxidized sodium alginate (OSA) retains the water solubility and biocompatibility of alginate, while acquiring better biodegradability and molecular flexibility. In addition, it demonstrated ability to bind to collagen [20,22,23,24,25,26,27,28].

The industrial use of periodate is currently limited by its relatively high cost, being only reluctantly used in applications on a large scale. Periodate may cause severe skin burns, eye damage, and organ damage in case of prolonged exposure; furthermore, it is very toxic to aquatic organisms and may cause fire or explosion as from its ECHA registration dossier (ECHA—the European Chemicals Agency implements the EU’s chemicals legislation to protect your health and the environment) [29]. Therefore, according to the restrictive EU regulations on the use of chemicals (REACH regulation), Waste Framework Directive 2008/98/EC, Water Framework Directive 2000/60/EC, Industrial Emissions Directive 2010/75/EU and the Circular Economy Package, the use of periodate must be limited [30]. On the other hand, periodate combines high oxidation rates with optimized resource efficiency, thereby increasing the sustainability and industrial relevance of the process. In fact, it was reported that optimized setup could provide a technically robust, economically acceptable, and environmentally tolerable basis for the production of dialdehyde cellulose on a larger scale using periodate, for several biorefinery scenarios in the pulp and paper industries [31]. Very recently, a robust and self-cleaning electrochemical synthesis for the preparation and regeneration of periodate has been reported, allowing for lower costs for the use of periodate in the synthesis of regulated products [32].

### 1.3. Nano-Hydroxyapatite as Less-Toxic Additive for Improving the Flame Retardancy of Leather

Nanomaterials, due to their ability to penetrate into fibers and interact with collagen, are excellent candidates for purposely modifying collagen, i.e., by increasing the hydrothermal stability of the leather [33,34] and improving its technological properties [35,36]. Nano hydroxyapatite (nano-HAp) is among the simplest materials to synthesize, starting from available and economical raw materials. Hydroxyapatite (Ca_10_(OH)_2_(PO_4_)_6_), a well-known member of the calcium phosphate family with high biocompatibility, is the major inorganic component of bone and teeth in vertebrates. Hence, hydroxyapatite materials have been extensively investigated for applications in biomedical fields [37,38,39,40,41,42]. Recent studies on hydroxyapatite show its effect on fire resistance and smoke suppression of polyurethane fire-retardant coating [43,44]. Novel bio-based flame-retardant composites containing nano-HAp have been recently synthetized [45,46,47,48]. Although hydroxyapatite has been used for imparting flame retardancy to several polymers it has never been tested as leather flame retardant. Hydroxyapatite-based bio-nanocomposites were used as alternatives to conventional tanning and re-tanning chemicals to produce leather with higher thermal stability and tear strength [33]. The suitability of nano-HAp for use in the tanning process will enable substituting some of the current syntheses of flame retardants, and especially brominated flame retardants which were shown to be persistent in the environment, bioaccumulative in wildlife and humans, and toxic to laboratory animals and wildlife, producing reproductive, developmental, and systemic effects in lab tests. Their use has been restricted from July 2006 by the Restriction on Hazardous Substances (RoHS) Directive implemented by EU. An advantage of nano-HAp over other traditional flame retardants is its inherent compatibility with the main leather component (i.e., collagen) which is expected to trigger interactions between leather and nano-HA, which fixe the nanoparticles in the leather structure. As a result, there would be less volatilization and leaching, which are typical of conventional flame retardants, such as brominated ones, that are not chemically bonded to the objects they are supposed to protect [49].

In the present study, the tanning ability of OSA, as well as the ability of nano-HAp to enhance leather flame resistance were tested. The tanning process was developed in laboratory and at pilot-scale to test the process’s upscaling potential.

First, we investigated the interaction between OSA and collagen at a laboratory scale and optimized certain crucial variables from the standpoint of sustainability, including the quantity of NaCl and KIO_4_. Investigations were also performed focusing on the relationship between the collagen-OSA matrix and the nano-HAp. With the ultimate goal of manufacturing high-quality and reproducible leather, the process was shifted to pilot scale in the second step to identify and address variation between the lab and pilot scale.

Utilizing a variety of analytical techniques, the different levels of collagen structure in hide biomatrix and leather chemical matrix were targeted. At the mesoscopic, macroscopic, and molecular levels, the ability of OSA to interact with collagen and increase its hydrothermal stability has been assessed using the micro-DSC technique, and standard SR EN ISO 3380-2003 method, respectively [48,49]. The oxidation reactions on the -OH groups at the C-2 and C-3 locations of the uronic units of sodium alginate have been identified using the FTIR-ATR technique, and the interaction between nano-HAp and the collagen-OSA matrix has also been demonstrated by FTIR-ATR and SEM-EDS. Understanding water binding and molecular constraints in the collagen-OSA and collagen-OSA-nano-HAp matrices was achieved through the use of unilateral nuclear magnetic resonance (^1^H unilateral NMR) approach. ^1^H Unilateral NMR, also called NMR-MOUSE^®^ is a portable non-invasive NMR technique that allows measurements to be performed in non-invasive way without any sampling. The magnetic field is applied to one side of the object; therefore, measurements can be performed directly on large objects fully preserving the integrity and the dimension of the object under investigation. ^1^H unilateral NMR has been previously used for studying leather and parchment [50,51,52], plastic and polymers [53], object belonging to cultural heritage [50,53]. In order to demonstrate the uniformity of nano-HAp distribution throughout the leather structure, SEM-EDS analysis was also conducted. The fire test for aircraft materials was used to evaluate the fireproofing that nano-HAp imparts. Comparing the physical, chemical, and mechanical characteristics of commercial poly-aldehyde-tanned leather to OSA-tanned leather allowed for discussion. 

## 2. Materials and Methods

Calf hides (pelts) were made available by the Leather and Footwear Research Institute (ICPI) of the National Research and Development Institute for Textiles and Lather (INCDTP), Bucharest. Analytical-grade sodium alginate, potassium periodate and ethylene glycol were procured from Sigma Aldrich (Burlington, MA, USA). Industrial grade sodium alginate and ethylene glycol were purchased from Brenntagg SpA. Sodium chloride, sodium bicarbonate, and water used were industrial grade (provided by ICPI). The synthetic tannins used for comparison were glutaraldehyde (GA) (laboratory tests) and a commercial polyaldehyde (PA) (pilot tests). The pilot-scale synthesis of nano-HAp was developed by University of Turin and Kemia Tau within the M-Eranet project InSuLa (innovative materials and technologies for sustainable leather manufacturing for automotive) [48,54]. Calcium hydroxide (grade 96%), phosphoric acid (grade 85%), and ammonium hydroxide solution from Sigma Aldrich were used for the nano-HAp laboratory scale synthesis, while industrial-grade hydrated lime, phosphoric acid 85%, and NH_3_ solution 33% were used for industrial pilot synthesis.

### 2.1. Sodium Alginate Oxidation

Periodate easily oxidizes the hydroxyl groups on carbons 2 and 3 of the repetitive uronic units of sodium alginate leading, by the rupture of the carbon–carbon bond, to the formation of two aldehyde groups in each oxidized monomeric unit (Figure S1). We applied the oxidation procedure reported by Ding et al. [12,55] to obtain oxidized sodium alginate (OSA) in solution and used it to directly tan un-pickled pelt. The laboratory process involved adding potassium periodate to a mixture of 20 g of sodium alginate and 1000 mL of distilled water, stirring it in the dark for 6 h at room temperature, and then letting it sit overnight to allow oxidation. To obtain OSA with various oxidation degrees (ODs), two molar ratios between the monomeric unit of SA and KIO_4_ were used, precisely 1:0.8 and 1:0.2. After 24 h, the process was stopped by adding ethylene glycol in the same molar ratio as potassium periodate and continuing to stir for an additional 30 min. At pilot scale, only OSA with a low OD was prepared. The homogenous transparent OSA solution with a pH of 5.0 to 5.5 was used in the tanning process without further purification. This decision was motivated by industrial applicability. The OSA solution produced at the pilot scale was tested for shelf stability by holding it at room temperature for 60 days. There was no yellowing effect or phase separation, demonstrating the suspension’s stability.

### 2.2. Nano-Hydroxyapatite Synthesis

The synthesis of nano-HAp at laboratory scale was performed according to literature [48,54,56]. Diluted phosphoric acid (H_3_PO_4_) was dropwise added to a suspension of calcium hydroxide under stirring.

The reagents were combined in a 10:6 molar ratio, giving a typical stoichiometric neutralization reaction:

10Ca(OH)_2_ + 6H_3_PO_4_ → Ca_10_(PO_4_)_6_(OH)_2_ + 18H_2_O

In particular, 0.1 mol of calcium hydroxide (Ca(OH)_2_) was stirred for 20 min in 200 mL of distilled water, 0.06 mol of phosphoric acid (H_3_PO_4_) diluted in 200 mL of distilled water was added dropwise to the suspension over 45 min. The pH was maintained above 10.5 by addition of ammonium hydroxide solution. After completing the phosphoric acid addition, the suspension was left under stirring for 2 h. After this period, stirring was stopped and the suspension was left overnight in the mother solution to improve the gradual incorporation of calcium into the crystalline structure in order to bring the material’s Ca/P molar ratio closer to the stoichiometric value. This maturation phase also changes the shape of the crystals from needle-like to more blocky [57]. The suspension was then centrifuged (4000 rpm, 5 min) to separate the synthesized nanoparticles from supernatant. To remove the unreacted reagents, the particles were re-dispersed in water and centrifuged three more times at 4000 rpm for 5 min each. The nanoparticles were then dried overnight at 50 °C in an oven to remove residual water. The finished product had a 99.6% yield. Hydrochloric acid (HCl, Sigma-Aldrich, 37%) was added to the washing waters to neutralize the non-reacted ammonium hydroxide.

The same synthesis process, except for the drying step, was reproduced in batches from 5 kg to 100 kg at Kemia Tau, as previously mentioned by Ingrao et al. [48]. Suitable reagents for industrial manufacturing were selected as reported in the SI (Table S1 and Figures S2 and S3). In order to assess the formation of nanoparticles of hydroxyapatite, FTIR-ATR (Figure S4A) and XRD (Figure S4B) analyses were performed. Rod-like shape with an average length of approximately 100–150 nm and a tendency to form tight agglomerates was evidenced by SEM (Figure S5).

To avoid further agglomeration that can damage the leather surface by scratching, nano-HAp was used in suspension. Because of its simplicity and low cost of implementation, wet chemical precipitation is the most often utilized approach. It has been shown that some HAp nanoparticles could be cytotoxic depending on their characteristics and synthesis procedure [58]. A recent research has demonstrated that commercial rod-like shape nano-HAp with an average length of approximately 20 to 40 nm and a tendency to form big agglomerates, synthesized by wet chemical precipitation at room temperature, have excellent cytocompatibility and no genotoxicity [58].

### 2.3. Analyses Methods and Techniques

The shrinkage temperature of calf leather was measured using the standard method SR EN ISO 3380-2015 and a Giuliani Apparecchi Scientifiici apparatus with visual detection. This method allows to identify the temperature at which the collagen fibers are massively destroyed and cause a visible contraction of a leather strip sample of (1.5 × 5) cm heated in water at a rate of 2 °C/min [59]. The temperature measuring device is graduated to 1 °C and shown to be accurate to ±0.5 °C. The detection precision and reproducibility depend on the analyst’s visual sensitivity, but accuracy is enough for the needs of tanners. Each reported value represents an average of three tests on fresh samples. For all samples, the standard deviation was less than 2.

The hydrothermal stability of calf hides/pelts and tanned samples was measured using a high-sensitivity SETARAM Micro-DSC III calorimeter in the temperature range of (25–85) °C, at a 0.5 K min^−1^ heating rate, using 850 μL stainless steel (Hastelloy C) cells as previously described [50,51]. The low-scan rate ensures the equilibrium condition for DSC analysis and allows for accurately measuring the collagen denaturation parameters. Samples of about (5.0–10.0) mg were suspended in 0.5 M acetate buffer (pH = 5.0) directly in the measure cell and left for 30 min to assure their full hydration and avoid temperature and enthalpy variations with lower hydration levels. Experimental DSC data acquired with the SETARAM SetSoft2000 software were analyzed using PeakFit 4.1 (Jandel Scientific, Corte Madera, CA, USA). DSC multiple peaks were deconvoluted using the PeakFit asymmetric Gaussian fit function to improve the fit of the asymmetry in the peaks.

Due to material homogeneity, two measurements were sufficient to yield precise denaturation parameter values. Each reported value represents an average of two tests on fresh samples. Certified reference materials such as naphthalene, benzoic acid, and gallium were used to check the supplier calibration constants in the working temperature region. Several melting runs performed under the same experimental conditions as those performed for leather samples showed an agreement with the IUPAC recommended values [60] of 0.05% for temperature and 0.25% for enthalpy. 

The relaxometric behaviour of the samples obtained at laboratory was measured using similar ^1^H unilateral NMR devices (NMR-MOUSE^®^). In these types of instruments, the magnetic field penetrating the object is rather homogeneous and decreases with the distance from the surface of the magnet, therefore the NMR signal decays very quickly and must be recovered as an echo. Although the inhomogeneity of the magnetic field, the spin-lattice (T1) and the spin-spin (T2) relaxation time experiments give information on the chemical–physics properties of the material. An NMR MOUSE^®^ PM2 from Magritek GmbH controlled by a Kea 2 spectrometer operating at 27 MHz ^1^H resonance frequency was used for measuring the proton relaxation times for the laboratory samples [52] while an NMR MOUSE from Bruker Biospin interfaced with a single-sided sensor by RWTH, Aachen University, operating at 13.62 MHz to analyze the samples obtained at pilot scale [61].

In the first case, the ^1^H spin-spin (transverse) relaxation times T_2_ were measured using the Carr-Purcell-Meiboom-Gill (CPMG) pulse sequence with an echo-time (TE) of about 25 μs. The proton spin-lattice (longitudinal) relaxation times T_1_ were measured with the saturation-recovery pulse sequence using a Hahn-echo with an echo time of about 25 μs for detection. The analysis of the saturation recovery data was best performed with the help of a single exponential function.

(1) $$A(t)=A_{\mathrm{equilibrium}}(1-exp(-\frac{t}{T_{1}})),$$

In the second case, the longitudinal relaxation times T_1_ were measured with the saturation–recovery pulse sequence followed by a CPMG-train in the detection period to increase the sensitivity (32 echoes). Transverse relaxation times T_2_ were measured with the CPMG sequence, and 256 echoes were recorded with an echo time 2τ of 42 μs, at a depth of 3 mm. The data obtained applying the CPMG pulse sequence were fit to the following function:

(2) $$Y=\sum_{i=1}^{n}W_{i}e^{\frac{-t}{T_{1i,2i}}},$$

where *n* is the number of components used to fit the decay of the magnetization, *W_i_* is the spin population of the ith component, and T_1i,2i_ is the longitudinal or transverse relaxation time of the ith component. The sum of spin populations was normalized to 100%.

With both instruments, the error in the reported T_1_ and T_2_ values was within 10% of the nominal values.

FTIR-ATR analyses were carried out on grain, corium, and cross-sections of the samples, using an Alpha spectrometer (Bruker Optics) equipped with a Platinum ATR module. Spectra were recorded in the 4000–400 cm^−1^ spectral range with a 4 cm^−1^ resolution, using 32 scans. The OPUS 7.0 software was used for processing and evaluating spectra. 

SEM images were acquired using a SEM ZEISS (EVO50 XVP) instrument. The accelerating voltage (EHT) has been set between 10 and 15 kV, with a resolution of 10 nm and a LaB_6_ source. The leather samples were preventively cut into pieces of 1 cm × 1 cm and deposited on stubs with a double-sided carbon tape in order to promote conductivity. For the same reason, the samples have been subsequently subjected to metallization by the deposition of a thin layer of gold (ca. 15 nm) using a specific sputter coater.

## 3. Results and Discussion

### 3.1. Laboratory-Scale Tanning Test Using OSA and Nano-HAp: Study of Their Interaction with Collagen Using Micro-DSC, NMR-MOUSE, ATR-FTIR and SEM-EDS

It is worth mentioning that, although its limitation is desirable, NaCl has a crucial role in the tanning process. It disturbs the water layers of collagen-water hydrogen bonds [62], improving the opening up of collagen fiber network and enhancing porosity, as well as exposure and ionization of charged side-chain groups in collagen [63]. In addition, NaCl weakens the strong electrostatic interaction between collagen and OSA, an anionic polysaccharide. NaCl ions shield the charge of polyelectrolytes in solution, disfavoring the electrostatic interactions, and then, the importance of non-electrostatic forces on OSA-collagen interaction increases [64,65]. The use of a limited, but adequate amount of NaCl is therefore peremptory.

The ability of OSA to act as a tanning agent was tested depending on both the OD and NaCl amount in the tanning bath. Initially, a comparative study was carried out using OSA with a higher OD and halving the concentration of NaCl from 12% to 6%. A reduction of NaCl amount is in fact highly desirable due to the difficulty of removing it from the final tanning liquor [50,51]. In a second step, the ability of OSA with low OD to tan when NaCl amount was reduced by half was tested. The tanning process is described in the SI (Table S2).

#### 3.1.1. Hydrothermal Stability of Collagen-OSA Chemical Matrix by Micro-DSC

The most important change induced by the tanning process concerns with hydrothermal stability increase due to collagen–tannin chemical interaction. According to Covington, the tanning action of any chemical species is manifested by the creation of a collagen-tannin chemical matrix which acts as a single chemical compound, whose stability depends on the nature and interactions of collagen, water, tanning agent, or the main tanning agent and the counterion or secondary tanning agent [66].

The hydrothermal stability of the pelts treated with OSA with different OD in the presence of different amount of NaCl was evaluated both at the material level (using the standard method SR EN ISO 3380-2003.13) and at the fibrillar level (using micro-DSC technique) [51,67]. The values of shrinkage temperature *T*_s_ of collagen fibres measured by the standard method are presented in Table 1 together with the denaturation parameters of collagen fibrils obtained by micro-DSC analysis. 

SR EN ISO 3380-2003.13 is a visual test conducted in current tanning practice to evaluate the effectiveness of the tanning process. It measures the shrinkage temperature of a leather sample with well-defined dimensions heated slowly (2 °C/min) in water. *T*_s_ is the temperature at which a leather sample suddenly shrink. The higher the shrinkage temperature, the better the moist heat resistance of the leather.

The hydrothermal stability is accurately measured by micro-DSC, a highly sensitive technique very little used in evaluating the effectiveness of the tanning process [68], but often used to determine the thermal stability of proteins. It directly measures the variation of a thermodynamic property of materials, the specific heat capacity *C*_p_, as a function of temperature. *C*_p_ measures how the material stores additional energy at the molecular level as it is heated. *C*_p_ is a fundamental, thermodynamic property of a material and is the best way to compare samples. The *C*_p_ measurement during a micro-DSC experiment show the amount of energy required to increase the sample’ temperature and make it go through a thermal transition visible as a peak. In our specific case, the thermal transition is collagen matrix denaturation. The total energy required to heat and denature a quantity of leather, is called denaturation enthalpy Δ*H* and it is calculated as the integral of heat capacity function over the temperature range (corresponding to peak area). Denaturation enthalpy is an extensive parameter directly related to the quantity and strength of the forces (covalent and non-covalent interactions) which stabilizes the collagen-tannin matrix. The maximum temperature on a *C*_p_ profile (peak) is called *T*_max_ and it considered the statistical temperature of denaturation when folded (native) and unfolded (denatured) collagen are present at equal concentrations. *T*_max_ is shifted towards the higher temperatures when the collagen-tannin matrix stability increases. Onset temperature *T*_onset_ is defined as the intersection of the tangent of the peak and the extrapolated baseline. *T*_onset_ is more reliable and repeatable than peak temperatures, especially in the case of broad, multicomponent peaks. It indicates the structural destabilization of the collagen population with the lowest hydrothermal stability occurring prior to denaturation [50]. The half width of the peak Δ*T*_1/2_ is the full width at half maximum of the denaturation peak and gives a measure of the breadth of the distribution of molecular thermal stabilities within collagen chemical matrix. The Δ*T*_1/2_ value directly correlates with the degree of homogeneity of the tanning process [50].

**Figure 1:**
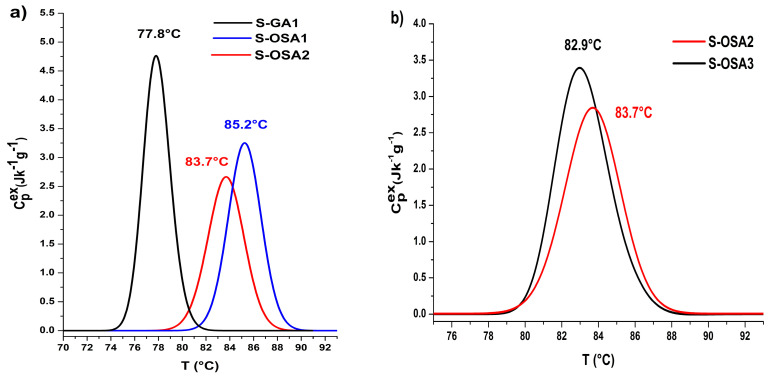
Micro-DSC denaturation peaks of samples tanned with (**a**) GA (black) and 12% NaCl; OSA1 (SA:KIO_4_ molar ratio 1:0.8) and 12% NaCl (blue); OSA2 (SA:KIO_4_ molar ratio 1:0.8) and 6% NaCl (red). (**b**) OSA2 (SA:KIO_4_ molar ratio 1:0.8) and 6% NaCl (red); OSA3 (SA:KIO_4_ molar ratio 1:0.2) and 6% NaCl (black).

Although the mechanism underlying the increase in hydrothermal stability through tanning is not yet fully understood, shrinkage temperature, and thermodynamic parameters of collagen-matrix denaturation are most useful for distinguishing between different classes of tannins [61,69,70]. In this particular case, they allowed us to compare the tanning ability of OSA and commercial aldehyde-based tannins (Figure 1, Table 1).

The first finding from the data in Table 1 relates to the significantly higher hydrothermal stability of the collagen matrix in OSA-tanned samples as opposed to glutaraldehyde-tanned one. This behavior supports the formation of reactive aldehyde groups along the alginate backbone with greater rotational mobility and the formation of an intermolecular network of *hydrogen bonds*. The peak half width increase may be explained by OSA’s variable molecular mass. The very slight differences between the values of the denaturation enthalpy of the glutaraldehyde-tanned and OSA-tanned samples suggest both tannins appear to have the same type of chemical interactions with the collagen in the collagen matrix [71].

By halving the NaCl concentration, the values of the denaturation temperature, onset temperature, and denaturation enthalpy decreased, while the peak half-width value slightly increases. These effects most likely come from a little less effective OSA penetration within the hide structure due to a decreased swelling of the fibrous collagen network. At the material (macroscopic) level, the effect on the thermal stability of the fibrils caused by lowering the NaCl concentration is almost not discernible, since the shrinkage temperature *T*_s_ for the S-OSA1 and S-OSA2 samples were very similar. As a result, the swelling produced by a 6% NaCl tanning bath is considered to be satisfactory for the tanning process, with the added benefit of lowering the environmental impact of tannery effluents. At both the material (*T*_s_) and fibril (*T*_max_) levels, a relatively small loss in thermal stability was seen as the result of the lowering of the SA:KIO_4_ molar ratio from 1:0.8 to 1:0.2. This outcome permits the lowering of the amount of KIO_4_ and, consequently, ofOD of OSA, without altering the macroscopic thermal stability of leather.

#### 3.1.2. ^1^H Unilateral NMR and FTIR-ATR Analysis of Collagen-OSA Chemical Matrix in Leather

To support the calorimetric data, the relaxometric characterization of tanned samples was carried out (Table 2). According to a previous study, both the transversal relaxation time T_2_ and longitudinal relaxation time T_1_ are sensitive indicators of the condition of materials [53]. The monoexponential component T_1_ has comparable values for all samples (≈30 ms), indicating a similar aldehyde-bonding mechanism in both the S-GA1 and S-OSA2/3 samples (Figure S4). In fact, it was previously reported that the T_1_ value of leather is a measure of the strength of water-mediated bonding in the collagen matrix, depending on the number of sites capable of strong interactions with water, which, in turn, depends on both the chemical structure of tannin and micromorphology of collagen in the animal hide [72].

Unlike T_1_, the transversal relaxation times T_2_ for S-OSA samples obey to a multi-exponential relaxation, providing two different components: a short relaxation time (T_2A_), in the range of (0.17–0.30) ms, and a long relaxation time (T_2B_), in the range of (0.5–1.11) ms. In the case of S-GA1, there is also a third component with a longer T_2_ value (T_2C_ = 15 ms), in small percentage. As the GA is a commercial product, the third T_2C_ component can be explained by the presence of additives such as mineral oils, sulphates, acids.

**Figure 2:**
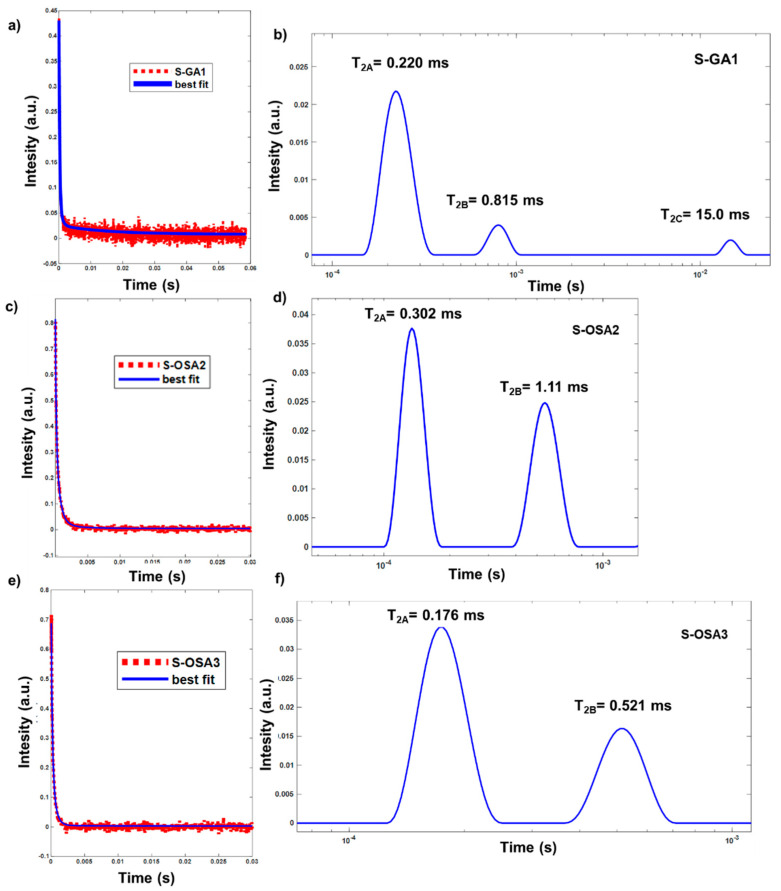
^1^H CPMG decay curves (**a**,**c**,**e**) and transversal relaxation time (T**_2_**) distribution (**b**,**d**,**f**) calculated with the inverse Laplace transform for S-GA1, S-OSA2 and S-OSA3.

Water is a fundamental component of the collagen hierarchical structure, and several authors have suggested that the short component of the transversal relaxation time is caused by water molecules tightly bound to collagen helices in the interfibrillar space whereas the long component is caused by water molecules bound to microfibrils [64,65,67,68]. As a result, the long component is significantly more mobile, sensitive to the effects of tanning agents, and vulnerable to variations in hydration levels [66] than the short component. Van Stiphout claimed that the short and long components of T_2_ are connected to more or less rigid collagen matrix structures, i.e., short relaxation times correspond to rigid (tightly bound) structures, while long relaxation times correspond to more loosely bonded structures [73]. Accordingly, the increase of the percentage of W_B_ corresponding to the looser bonded structures (Table 2 and Figure 2) in S-OSA3 (32%) compared to S-OSA2 (14%) and S-GA1 (12%) can be interpreted in terms of an increased chain mobility and reduction of tightly bonded structures [74]. This suggest a less densely packed collagen matrix and could be explained by the higher molecular mass distribution and lower amount of aldehyde groups of low OD OSA [55].

It is important to note that the NMR relaxometric parameters and the micro-DSC data have a good correlation. While the rise in OSA molecular mass distribution corresponds to peaks with wider widths, T_1_ values closely match the decrease in thermal stability that *T*_s_ and *T*_max_ data indicate: S-OSA2 > S-OSA1 > S-GA1.

**Figure 3:**
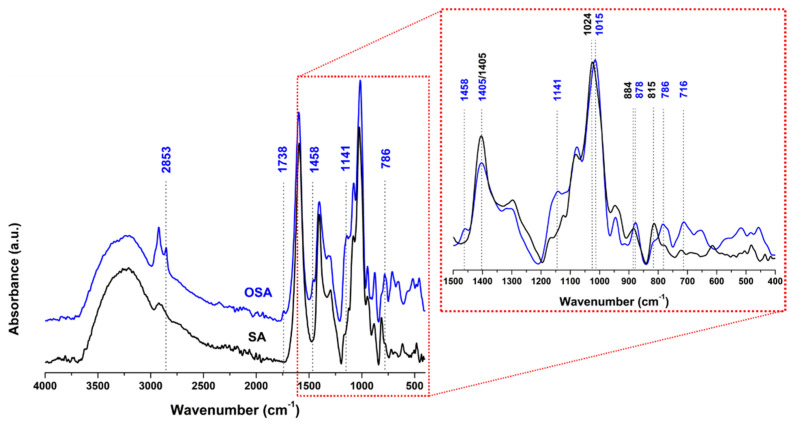
Comparison of ATRFTIR spectra of SA and OSA.

ATR-FTIR analysis was also performed to further confirm the formation of free reactive aldehyde groups in the OSA chain, as well as the interaction between OSA and collagen (Table S3). In Figure 3, a faint new peak at 1734 cm^−1^ is observed in the OSA spectrum and assigned to the vibration of the C=O bond of the aldehyde group [14,75]. There are also spectral changes in the symmetric C-O-C region of OSA spectrum; the signals at 884 cm^−1^ and 1024 cm^−1^ in the SA spectrum are shifted to 887 cm^−1^ and 1015 cm^−1^ in OSA spectrum. In addition, the decrease of the signal at 815 cm^−1^ can be attributed to the C-O-C decomposition on the alginate chains after oxidation [76]. A new band at 1141 cm^−1^, corresponding to the antisymmetric C-O-C mode, was observed in the spectrum of OSA [14].

**Figure 4:**
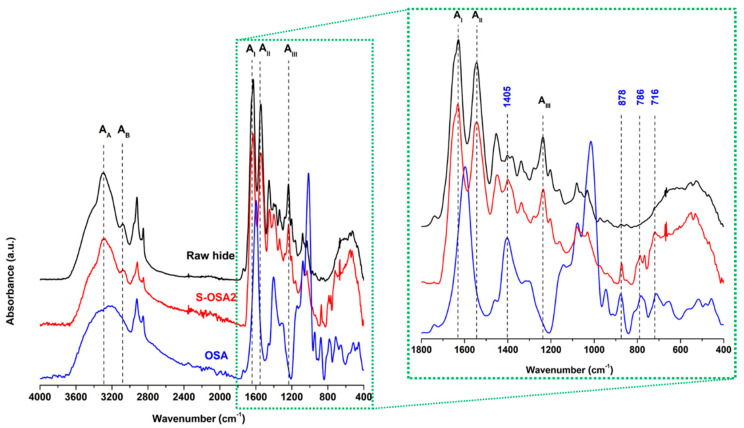
ATR-FTIR spectra of raw hide (black), S-OSA2 (red), and OSA (blue) in the 4000–400 cm^−1^ region. The region 1800–400 cm^−1^ is highlighted. Amide I, amide II, amide III, amide A and amide B bands of collagen are highlighted.

Figure 4 suggest that the backbone of collagen in the presence of OSA does not change because the positions and intensities of main amide bands still maintain, especially the amide I, II, and III bands correlated to the helix structure of collagen [76,77]. Moreover, OSA-tanned leather shows the characteristic peaks of OSA, confirming the formation of a collagen-OSA matrix: the band at 1405 cm^−1^, attributable to the symmetric stretching vibrations of carboxylate groups (COO^−^) of the polymeric backbone of alginate [78] and the signals of the alkyl groups of OSA occurring at 786 cm^−1^ and 716 cm^−1^ [79]. The presence of the characteristic band of hemiacetal at 878 cm^−1^ in the spectrum of OSA-tanned leather could be considered proof of coupling reaction between the aldehyde groups of OSA and amine groups of collagen.

The results obtained at laboratory level confirmed the ability of OSA with low oxidation degree to interact with collagen and provide thermal and structural stability to leather collagen matrix, comparable to that provided by the commercial glutaraldehyde. We therefore moved on to the next step, namely the introduction of nano-HAp in the post-tanning process.

### 3.2. Laboratory Scale Tanning Test with OSA as Tanning Agent and Nano-HAp Wet Treatment

**Figure 5:**
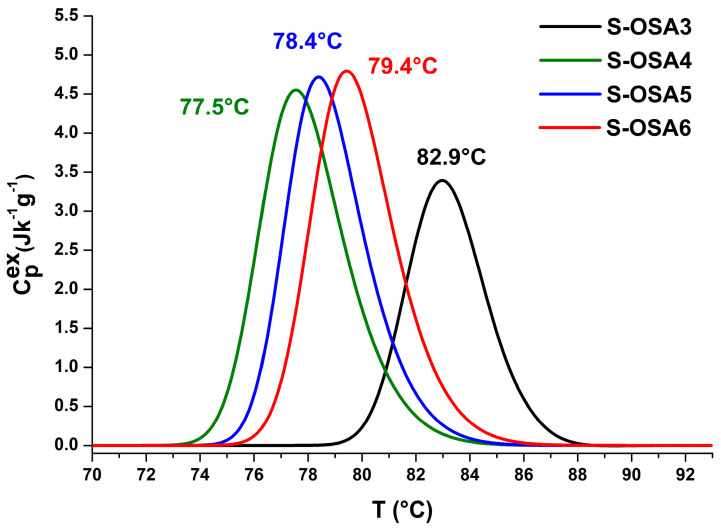
Micro-DSC thermal denaturation peaks of collagen matrix in the samples treated with OSA (black); OSA and nano-HAp 1.0% (green); OSA and nano-HAp 1.5% (blue); OSA and nano-HAp 3.0% (red).

To find the lowest nano-HAp concentration that guarantees a good flame-retardant effect without lowering the leather’s performance in terms of hydrothermal stability, low OD OSA, and nano-HAp suspensions with varied concentrations were utilized. The assessment of the hydrothermal stability of leathers treated with nano-HAp was performed by micro-DSC analysis (Table 3 and Figure 5).

The results shown in Table 3 indicate a partial de-tanning, as expected, as a result of the wet treatment with nano-Hap, evidenced by the slight decrease of thermal stability. A partial unbinding of the OSA molecules might occur due to competitive interactions with water and nano-HAp. On the one hand, water clusters form and expand close to the tannin binding sites causing partial de-tanning, and on the other hand, nano-HAp molecules interact with collagen. Even though the mechanism of the collagen–hydroxyapatite interaction is not yet known, recent ab initio simulations revealed relatively strong electrostatic interaction between the proline carbonyl C=O group and the most exposed Ca ion of the P-rich HAp surface [56,80,81]. In fact, the significant increase of the denaturation enthalpy of nano-HAp-treated samples confirms the formation of relatively strong interactions between collagen and nano-HAp. It is interesting to note that an increase in nano-HAp concentration simply attenuates the decrease in the denaturation temperature rather than increasing collagen matrix denaturation enthalpy. Depending on the nano-HAp concentration, this may be induced by varying levels of confinement within the fiber matrix, as observed by Miles and Avery for collagen in skin and decalcified bone [82]. Nano-HAp may alter the collagen’s intermolecular spacing, causing a different packing and, consequently, a different way of “boxing in” by surrounding molecules in the fiber matrix. This is in line with Miles and Ghelashvili’s “polymer-in-a-box” theory, which states that the collagen molecule is more thermally stabilized the smaller the “box”’s dimensions are—i.e., the closer together the collagen molecules are in the fiber [83]. This suggests that there are fewer possible configurations, activation entropy is less effective, and molecule thermal stability is better.

**Figure 6:**
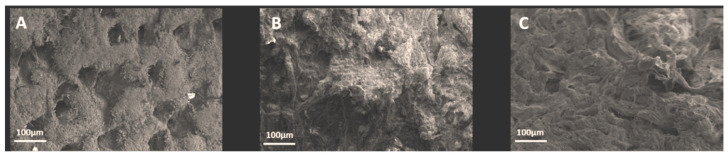
SEM images taken form S-OSA3: (**A**) grain, (**B**) corium, and (**C**) cross-section.

Both the grain (hair) and corium (flesh) sides of the samples, as well as the cross-section, were subjected to SEM-EDS observations. The complex micromorphology of the leather sample is illustrated in Figure 6 for the S-OSA3 sample. The pattern of hair holes is visible on the grain side, with some salt agglomerates trapped in the hair holes, and the typical mesh-like fibrous structure is visible in cross-section.

**Figure 7:**
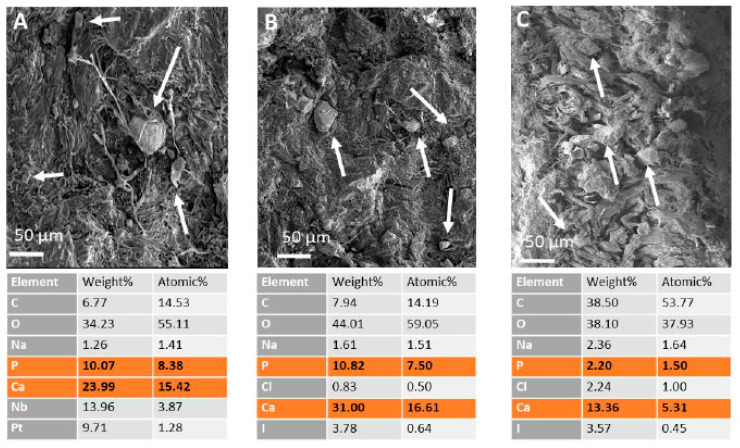
SEM images of OSA-tanned samples wet-treated with 1% of nano-HAp: (**A**) corium, (**B**) grain, (**C**) cross-section. For each image, the EDS analysis is reported in table. Nano-HAp bulky crystals are indicated by arrows.

In Figure 7, SEM images of the sample treated with 1% nano-HAp show the presence of solid agglomerates on both of the lateral sides (corium and grain) and in cross-section. EDS elemental mapping confirm the presence of nano-HAp both on the two lateral sides and in the skin structure.

**Figure 8:**
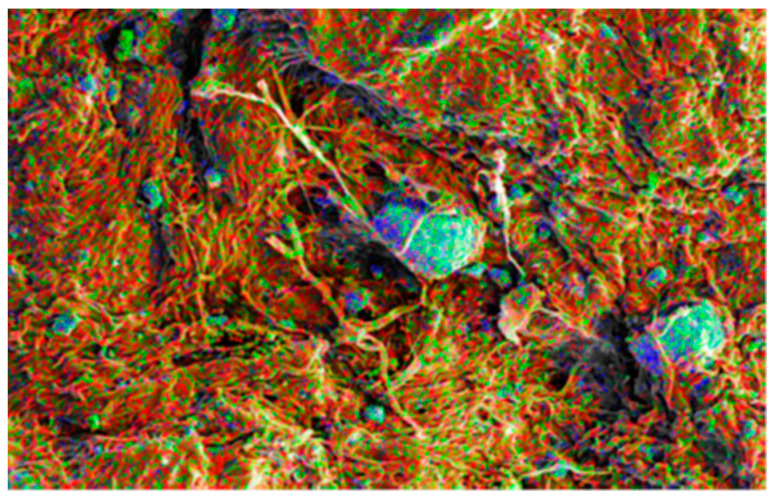
Elements map on SEM image of S-OSA4 (wet-treated with 1% nano-Hap). Color code: red is phosphorous, black is calcium, green is carbon, and blue is oxygen.

For the purpose of determining the distribution of nano-HAp, the EDS examination was carried out throughout the entire region, not just on the agglomerates. By representing each element with a distinct color, EDS element distribution mapping, which enables a qualitative estimation of the nanoparticles distribution, was used to analyze the homogeneity of nano-HAp distribution (Figure 8). It is clear that P and Ca, the two primary components of nano-HAp, are most abundant on the leather surface and are evenly distributed throughout (rather than just in agglomerates). But as was to be expected, more nano-HAp was observed on the surfaces than in the cross-section.

FTIR-ATR analysis was performed to monitor functional groups after the nano-HAp treatment and eventually find out evidence of its interaction with collagen-OSA matrix.

**Figure 9:**
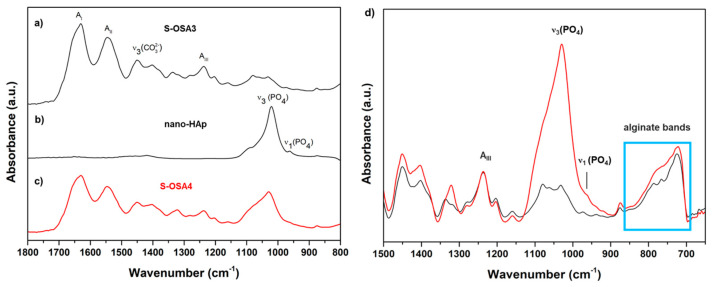
FTIR-ATR spectra of (**a**) S-OSA3, (**b**) nano-Hap, and (**c**) S-OSA4 in the 1800–800 cm^−1^ region. Those in (**d**) are reported the spectra of S-OSA3 (black) and S-OSA4 (red) in the 1500–650 cm^−1^ region. The blue box indicates the alginate fingerprint region and bands.

In Figure 9, the spectra (a–c) of nano-HAp, S-OSA3 (not treated) and S-OSA4 (wet-treated with 1% of nano-HAp) are reported, as well as the overlapped spectra (d) of S-OSA3 and S-OSA4. The common bands in the spectra of S-OSA3 and S-OSA4 samples are those typical to collagen, namely Amide I, Amide II, and Amide III bands. It is known that the ratio of the Amide I and Amide II relative intensities (AI/AII) is related to structural changes in the collagen molecule [84]. We calculated this ratio for the sample tanned with OSA, before and after treating it with nano-HAp. Since Amide I is affected by a huge contribution of water bending, the calculation was performed after the deconvolution of this band. The AI/AII ratio remained constant (≈1) for both S-OSA3 and S-OSA4, witnessing no interference of nano-HAp on the structural stability of collagen helical structure. Carbonate bands [85,86], due to the presence of calcium carbonate salts in leather samples, are detected at around 1400 and 870 cm^−1^, while the carbonate band around 700 cm^−1^ is hindered by the alginate bands [9]. The skeletal alginate fingerprint, or anomeric region, typical to carbohydrates, is detected at (950–750) cm^−1^ [86,87]. The typical signals of HAp, related to phosphate groups became visible in the region (1090–960 cm^−1^): the most intense peak at 1013 cm^−1^ corresponds to the stretching vibrations of PO_4_^3−^ in the apatite (Figure S3) [88,89]. Interestingly, at the lower-frequency region (850–700 cm^−1^), a modification in the alginate monomeric unit bands appeared after the treatment with nano-HAp: the two separate signals clearly visible S-OSA3 spectrum merge into a single band in the S-OSA4 spectrum. This might be attributed to the mineral and organic phase interaction.

### 3.3. From Laboratory to Industrial Scale: A Scale-Up Framework for a Tanning Process Using OSA and Nano-HAp

OSA and nano-HAp were obtained at pilot scale level as reported in materials and methods section. The industrial tanning process was developed starting from a wet-white technology already in use at Kemia Tau by substituting the commercial tanning agent (synthetic polyaldehyde) with OSA as reported in Table S4. Unlike the tests at the lab level, when the leathers were immersed in the nano-HAp suspension, the suspension of nano-HAp was applied using roll coater technology (Figure S7) [90]. With this method, the disadvantages of agglomerating nano-HAp particles and disposing of those that did not enter the leather at the end of the treatment are avoided. In addition, we were able to keep the concentration of nano-HAp to 1%.

To evaluate new leather prototype performance in terms of chemical stability, physical-mechanical qualities and fire resistance, it was compared to commercial leather both before and after the roll coating treatment with nano-HAp.

#### 3.3.1. Thermal Stability and Chemical Characterization 

**Figure 10:**
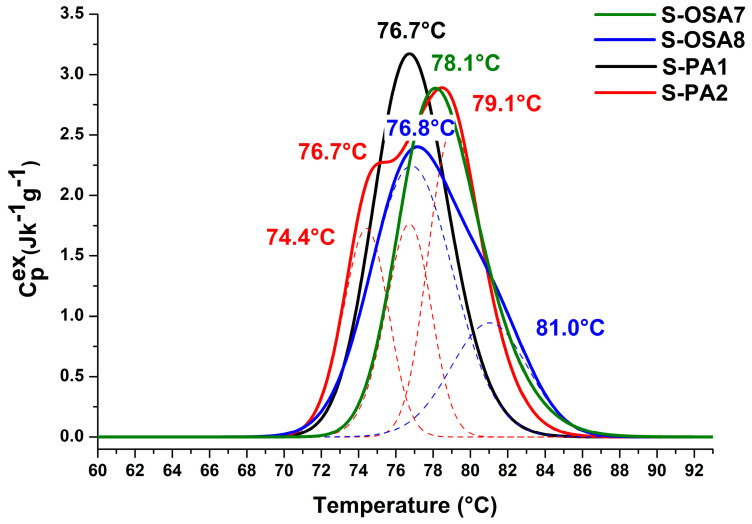
Micro-DSC denaturation peaks of new OSA-tanned leather and commercial leather before, S-OSA7 (green) and S-PA1 (black), and after the roll-coating treatment with 1% nano-hAp suspension, S-OSA8 (blue) and S-PA2 (red). Multicomponent denaturation peaks are deconvoluted into constituent peaks represented by dot lines.

The thermal stability of both new OSA-tanned prototype and commercial leather was evaluated by micro-DSC and the results are reported in Figure 10 and Table 4. The denaturation peaks of both OSA-tanned and commercial leather are monocomponent, sharp peaks with a maximum at 78.1 °C and 76.7 °C, respectively, showing similar values for the onset temperature, peak width, and denaturation enthalpy. Such a similar behavior suggests a similar confinement in the fiber matrix for most collagen molecules, that is similar tanning mechanisms. It also confirms the suitability of OSA as a wet-white tanning agent.

The DSC profiles of leathers changed in a similar way after being wet-treated with nano-Hap; the peaks broadened significantly and took on a multicomponent character. In fact, two populations of collagen can be observed, one of which is slightly more stable and the other slightly less stable than the collagen–OSA population present in the leather prior to the addition of nano-HAp. We could deduce from this behaviour that the collagen-OSA matrix and nano-HAp interacted to produce a more stable population that was mostly associated with the outer layers of leather (as suggested by EDS analyses). The partial release of tannin (either OSA or commercial tannin) during the wet treatment (which always occurs in the wet re-tannin process) may be responsible for the less stable collagen population. It is worth noting that the percentage of collagen interacting with nano-HAp is the same regardless of the tanning agent. These findings indicate that the collagen matrix’s thermal stability in S-OSA7 and S-PA1 (before nano-HAp treatment) is comparable, and that it is also comparable in S-OSA8 and S-PA2 (after nano-HAp treatment). 

**Figure 11:**
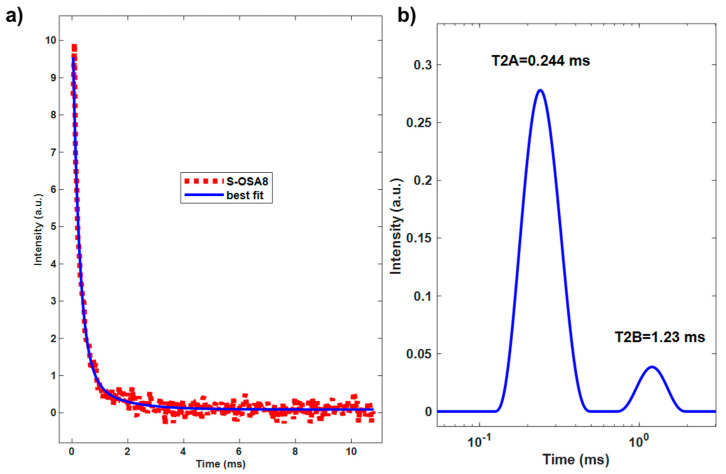
(**a**) ^1^H CPMG decay curve of S-OSA8 and (**b**) transversal relaxation time (T_2_) distribution calculated with inverse Laplace.

The innovative technology based on OSA and nano-HAp was also evaluated by assessing the relaxometric properties of leather, specifically the longitudinal and transverse relaxation periods of protons in the collagen matrix (Figures S8 and S9). The results from Table 5 and Figure 11, Figures S8 and S9 are in excellent agreement with those from the micro-DSC. The T_1_ value drop reflects the rise in matrix strength brought on by the interaction with nano-HAp. The close T_1_ values of S-OSA8 and S-PA2 support their similar thermal stabilities. The percentage W**_A_** of the T_2A_ short component increase after nano-HAp treatment indicates a decrease in molecular mobility. However, the concurrent decline in T_1_ and W**_B_** may be due to a reduction in the collagen matrix’s conformational flexibility brought on by a drop in the number of molecular configurations possible as a result of molecules’ bonds with nano-HAp.

Considering that the degree to which collagen fibres can swell and expand in a particular environment decreases as a result of the interaction collagen and nano-HAp, this also affects the denaturation temperature of fibres that are fully saturated with water (the condition in which the micro-DSC measurements are preformed). In general, fibres with varying linkages will equilibrate to different intrafibrillar fluid volumes and, as a result, have different temperature stabilities in a given environment. The stabilizing effect of nano-HAp could thus be explained by the stabilizing mechanism of the “polymer-in-a-box”. It relies on the idea that the stability of a collagen molecule in a fibre is made up of two terms: (i) the intrinsic stability of the molecule itself, without the stabilizing interactions of surrounding, and (ii) the stability gained by collagen–collagen interactions, i.e., the stability gained from being contained in the box [83]. The interaction between HAp nanoparticles and collagen could be explained in terms of new non-covalent bonding, i.e., electrostatic interaction. Earlier research demonstrated electrostatic contact between the proline carbonyl C-O group and the most exposed Ca ion of the P-rich HAP surface [81], as well as between the carboxylate groups of collagen and hydroxyapatite [86]. From a technological perspective, nano-HAp’s capacity to bind to collagen matrix enables the replacement of current flame retardants while enhancing the stability and strength of the collagen matrix.

**Figure 12:**
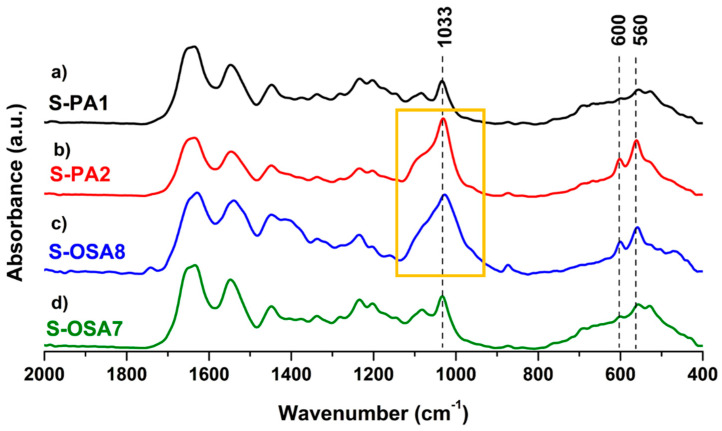
FTIR-ATR spectra of leather before (S-PA1 and S-OSA7) and after the treatment with nano-HAp (S-PA2 and S-OSA8) in the 2000–400 cm^−1^ range. The yellow box indicates the strong antisymmetric stretching of the phosphate group.

The intense bands of the phosphate group, i.e., antisymmetric stretching in the range (1090–960) cm^−1^ and bending at 560 and 600 cm^−1^ [88] (Figure 12) were found in both S-PA2 and S-OSA8 FTIR-ATR spectra, confirming the interaction between collagen and nano-HAp.

#### 3.3.2. Fire Resistance Characterization 

**Figure 13:**
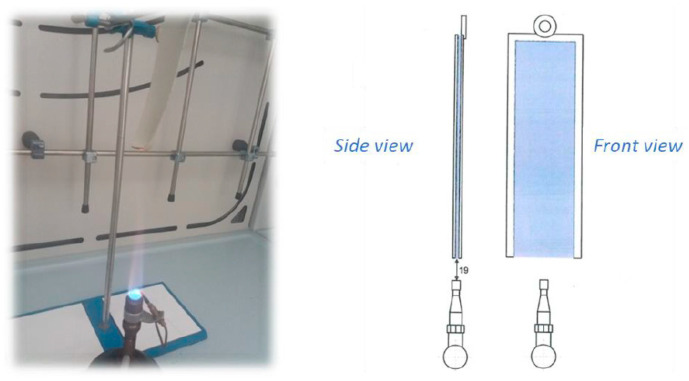
FAR/JAR 25.853 vertical test; leather sample placed vertically on a gas flame.

The evaluation of the fire performance of leathers treated with nano-HAp was carried out via the standard fire resistance test FAR/JAR 25.853 (vertical test) regulated by the Federal Aviation Administration (FAA) and by EASA (European Aviation Safety Agency). This method measures the flammability of the material exposed to a Bunsen burner flame by recording the flame propagation distance after a certain flame time (Figure 13).

**Figure 14:**
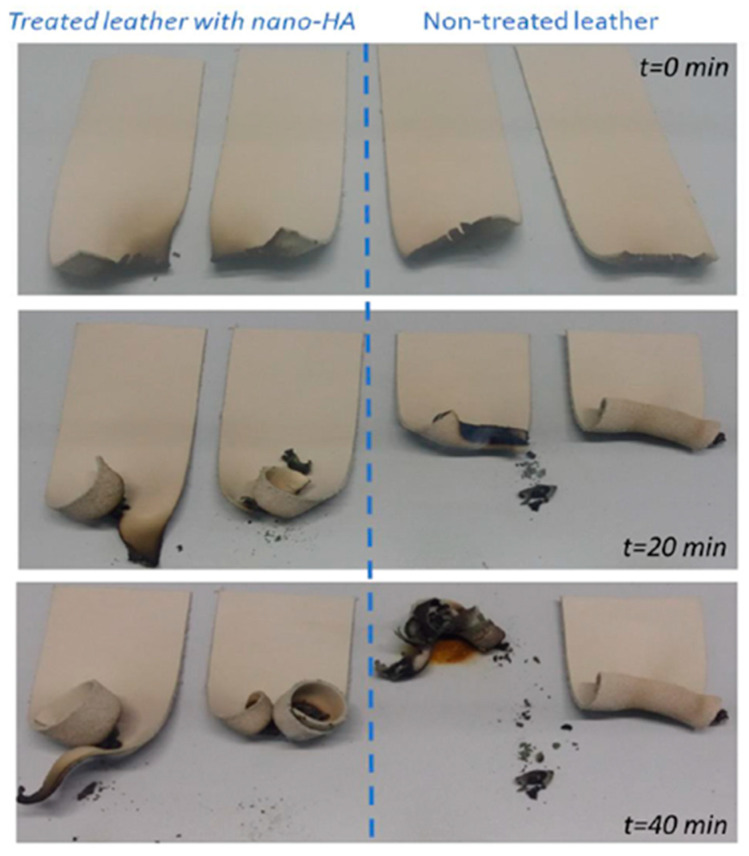
Leather samples treated with nano-HAp (on the left side) and not-treated (on the right side) subjected to FAR/JAR 25.853 (vertical) test. The photographs were taken at 0 min, 20 min, and 40 min following leather contact with a gas flame for 60 s.

The burning length and flaming time of drippings from the leather samples were measured during the test, which involved exposing the samples vertically to a Bunsen burner flame for 12 and 60 s (Figure 13). The fire behavior of leather samples (both those treated and those not treated with nano-HAp) is shown in Figure 14: the consumption of leather was measured across a range of times: 0, 20, and 40 min. Leather treated with nano-HAp demonstrated significantly improved fire resistance compared with non-treated leather; it suffers only partial combustion and the flame was quickly extinguished. This behavior demonstrates the impact on the fire resistance of leather of nano-HAp at a very low concentration that could be attributed to its great thermal stability and capacity to enhance char quality [43,91].

#### 3.3.3. Physical Mechanical Characterization

Physical–mechanical testing was carried out to confirm the new leather’s practical usefulness. According to the findings in Table 6, we can infer the following:

- In terms of tensile strength, tearing, and cracking resistance, S-OSA7 and S-OSA8 demonstrated behavior resembling that of commercial leathers.
- OSA-tanned leathers displayed superior deformation and elastic–plastic behavior.
- Nano-HAp treatment increased cracking resistance while slightly reducing tearing resistance.

**Table 6:** Physical–mechanical properties of new leather prototype compared to commercial leather obtained at pilot scale.

Test Name	Technical Characteristic	UM	S-OSA7	S-OSA8	S-PA1	S-PA2	Standard Method
Thickness	Thickness	mm	1.7	1.8	2.1	1.9	SR EN ISO 2589:2016 [92]
Tensile strength and percent elongation	Elongation at cracking	%	54.5	56.5	47.5	55.4	SR EN ISO 3376:2020 [93]
	Elongation at break	%	63.6	54.5	71.3	55.1	
	Tensile strength	N/mm^2^	18.7	12.3	20.3	12.0	
	Tear strength	N/mm^2^	12.9	13.7	13.3	13.8	
Tear strength in extension	Tear resistance	N	53.2	45.0	57.5	42.7	SR EN ISO 3377-1:2012 [94]
Tear resistance on two edges	Tear resistance	N	123.2	77.7	147.3	77.7	SR EN ISO 3377-2:2016 [95]
Softness	Ring opening Ø 20 mm	mm	2.6	2.3	3.0	1.9	SR EN ISO 17235:2016 [96]
	Ø 25 mm	mm	4.3	3.1	3.9	2.3	
	Ø 35 mm	mm	5.9	4.4	5.4	3.5	

## 4. Conclusions

We investigated innovative, commercially feasible, less-toxic, and biodegradable tanning (oxidized sodium alginate) and flame-retardant (nano-hydrohyapatite) agents in response to the pressing needs of the leather sector for the implementation of sustainable solutions. The suitability of sodium oxidized alginate (OSA) and nano-hydroxyapatite (nano-HAp) as a tannin and flame-retardant, respectively, for use in a traditional wet-white process, was proven by laboratory and pilot-scale tests. The analytical findings we obtained supported the usefulness and adaptability of OSA as a wet white tanning agent. Its tanning ability was also demonstrated by a four-fold decrease in the molar ratio of sodium alginate (SA) to potassium periodate (KIO_4_), from 1:0.8 to 1:02, even with a reduction in salt (NaCl) content compared to the conventional method. Consequently, the effluents will have lower NaCl concentrations, very little unreacted KIO_4_, and pH value that are easy to neutralize.

Micro-DSC, ^1^H NMR, and FTIR-ATR were used to investigate the tanning mechanism and collagen interaction with hydroxyapatite. Based on our findings and those published in the literature, we hypothesized that electrostatic interactions occurred during the interaction of nano-HAp with collagen matrix. The hydrothermal stability of OSA-tanned leather was comparable to that of commercial leather, whereas the nano-HAp treatment resulted in an overall increase in thermal stability and strength of collagen matrix, as evidenced by the occurrence of a more stable collagen population corresponding to nano-HAp-bonded collagen matrix. A good improvement of the fire-resistance time of leather was achieved using a very low concentration of nano-HAp (1%). The leather prototypes produced by tanning with OSA showed a physical–mechanical behavior resembling that of commercial leather.

In conclusion, our findings meet some of the most pressing demands of the leather industry, paving the way for a much more sustainable tanning process (metal-, formaldehyde-, and phenol-free), and biodegradable leather using bio renewable resources (sodium alginate), as well as a low-cost and simple method of preparing nano-Hap without requiring a significant change in current technology.

## Figures and Tables

**Table 1:** Hydrothermal stability parameters of the tanned samples: shrinkage temperature (*T*_s_) (measured by the SR EN ISO 3380-2003.13 method), denaturation parameters measured by micro-DSC: onset temperature (*T*_onset_), denaturation temperature (*T*_max_), peak half-width (Δ*T*_1/2_), and denaturation enthalpy (Δ*H*).

Sample Symbol	Tanning Agent	NaCl (%)	*T*_s_ (°C)	*T*_max_ (°C)	*T*_onset_ (°C)	Δ*H* (J/g)	Δ*T*_1/2_ (°C)
S-GA1	commercial glutaraldehyde (GA)	12	79	77.8	75.4	13.6	2.6
S-OSA1	OSA (SA:KIO_4_ molar ratio 1:0.8)	12	88	85.2	82.3	12.8	3.2
S-OSA2	OSA (SA:KIO_4_ molar ratio 1:0.8)	6	86	83.7	80.3	11.3	3.4
S-OSA3	OSA (SA:KIO_4_ molar ratio 1:0.2)	6	84	82.9	80.1	10.2	3.5

For denaturation parameters, each reported value represents an average of two micro-DSC runs on fresh samples, while for shrinkage temperature the standard deviation was less than 2 for all reported values.

**Table 2:** NMR-MOUSE relaxometric parameters of the samples tanned with OSA and commercial glutaraldehyde (GA).

Sample Name	Tanning Agent	T_1_ (ms)	W_A_ (%)	T_2A_ (ms)	W_B_ (%)	T_2B_ (ms)	W_C_ (%)	T_2C_ (ms)
S-GA1	commercial glutaraldehyde (GA)	30	85	0.220	12	0.815	3	15.0
S-OSA2	OSA (SA:KIO_4_ molar ratio 1:0.8)	34	86	0.302	14	1.11	-	-
S-OSA3	OSA (SA:KIO_4_ molar ratio 1:0.2)	31	64	0.176	32	0.521	-	-

The error in the reported T_1_ and T_2_ values is within 10% of the nominal values.

**Table 3:** The denaturation parameters of the collagen matrix in the samples tanned with OSA and treated with different percentages of nano-HAp (1.0, 1.5, 3.0%) measured by micro-DSC.

Sample Name	nHAp (%)	*T_onset_* (°C)	*T_max_* (°C)	Δ*H* (J/g)	Δ*T*_1/2_ (°C)
S-OSA3	0	80.1	82.9	12.8	3.5
S-OSA4	1.0	74.6	77.5	18.0	3.6
S-OSA5	1.5	75.6	78.4	17.4	3.3
S-OSA6	3.0	76.2	79.4	18.5	3.5

Each reported value represents an average of two micro-DSC runs on fresh samples.

**Table 4:** Micro-DSC denaturation parameters of new leather prototypes compared to commercial leather before and after nano-HAp treatment (pilot scale): onset temperature (*T*_onset_), denaturation temperature (*T*_imax_), peak half-width (Δ*T*_1/2_), denaturation enthalpy (ΣΔ*H*_i_), and the percentage of the various collagen populations (%Δ*H*_i_).

Sample Symbol	Tanning Agent/Nano-Hap	*T_imax_* (°C)	*T_onset_* (°C)	ΣΔ*H_i_* (J·g^−1^)	% Δ*H_i_*	Δ*T*_1/2_ (°C)
S-OSA7	OSA (SA:KIO_4_ molar ratio of 1:0.2)	*T*_1_ = 78.1	73.7	16.1	Δ*H*_1_ = 100	5.1
S-OSA8	OSA (SA:KIO_4_ molar ratio of 1:0.2) + nano-Hap (1%)	*T*_1_ = 81.0 *T*_2_ = 76.8	72.1	17.5	Δ*H*_1_ = 28 Δ*H*_2_ = 72	7.1
S-PA1	commercial poly-aldehyde (PA)	*T*_1_ = 76.7	72.7	18.1	Δ*H*_2_ = 100	4.7
S-PA2	commercial poly-aldehyde (PA) + nano-Hap (1%)	*T*_1_ = 79.1 *T*_2_ = 76.7	71.5	20.6	Δ*H*_1_ = 26.0 Δ*H*_2_ = 74.0	7.2

i = 1–2 represents the index of collagen populations within the analyzed samples. Each reported value represents an average of two micro-DSC runs on fresh samples.

**Table 5:** Longitudinal and transversal relaxation times of new leather prototype compared to and commercial leather—pilot scale.

Sample	Tanning Agent/Nano-HAp	T_1_ (ms)	W_A_	T_2A_ (ms)	W_B_	T_2B_ (ms)
S-OSA7	OSA (SA:KIO_4_ molar ratio 1:0.2)	34	86	0.302	14	1.11
S-OSA8	OSA (SA:KIO_4_ molar ratio 1:0.2) + 1% nHAp	25.4	92	0.244	8	1.23
S-PA1	commercial poly-aldehyde (PA)	29	83	0.205	17	0.756
S-PA2	commercial poly-aldehyde (PA) + 1% nHAp	26	89	0.236	9	0.854

The error in the reported T_1_ and T_2_ values is within 10% of the nominal values.

## Data Availability

Data are contained within the article and supplementary materials.

## Supplementary Materials

The following supporting information can be downloaded at: https://www.mdpi.com/article/10.3390/polym15244676/s1, Figure S1: Mechanism for the synthesis of OSA; Figure S2: nano-Hap production (5 kg and 100 kg); Figure S3: The thickening agents added to stabilize the formulation; Figure S4: FTIR-ATR spectra and XRD patterns of nano-Hap obtained at lab scale and industrial scale; Figure S5: SEM image showing the lamellar shape and dimension of the nano-Hap crystals Figure S6: Longitudinal relaxation time (T_1_) of S-GA1, S-OSA2 and S-OSA3; Figure S7: Representation scheme of the Roll Coater and scheme of the rotation of the gravure roller; Figure S8: Longitudinal relaxation time (T_1_) and transverse relaxation time (T_2_) of S-PA1, S-PA2, S-OSA7; Figure S9: Longitudinal relaxation time (T_1_) of S-PA1,S-PA2 S-OSA7, S-OSA8. Table S1: Reagents used for the preparation of nano-Hap at lab scale and at industrial scale, Table S2: Tanning process with OSA at laboratory level; Table S3: ATR-FTIR spectra of OSA and SA; Table S4: Tanning process with OSA at pilot-scale level.
