# Supplementary material for: Cleaner Leather Tanning and Post-Tanning Processes Using Oxidized Alginate as Biodegradable Tanning Agent and Nano-Hydroxyapatite as Potential Flame Retardant
Source: Polymers (Basel). 2023 Dec 11;15(24):4676. doi: 10.3390/polym15244676 (PMC10747597; doi:10.3390/polym15244676)
Supplement: Supplementary file 1 [file polymers-15-04676-s001.zip › polymers-2683360-supplementary.pdf]

Supporting information

Cleaner Leather Tanning and Post-Tanning Processes Using Oxidized Alginate as Biodegradable Tanning Agent and Nano-Hydroxyapatite as Potential Flame Retardant

Ilaria Quaratesi [1,†], Maria Cristina Micu [1,†], Erica Rebba [2], Cristina Carsote [3], Noemi Proietti [4], Valeria Di Tullio [4], Rita Porcaro [5] and Elena Badea [1,6,*]

[1] National Research and Development Institute for Textile and Leather (INCDTP), Research Institute for Leather and Footwear Branch (ICPI), Ion Minulescu Str. 93, 031215 Bucharest, Romania; i.quaratesi@gmail.com (I.Q.); 711mariacristina@gmail.com (M.C.M.)
[2] Department of Chemistry, NIS Interdepartmental and INSTM Reference Centre, University of Turin,
Via Pietro Giuria 7, 10125 Torino, Italy; erica.rebba@unito.it
[3] National Museum of Romanian History, Calea Victoriei Str. 12, 030026 Bucharest, Romania; criscarsote@yahoo.com
[4] Istituto di Scienze del Patrimonio Culturale (ISPC), Consiglio Nazionale delle Ricerche (CNR, Area della Ricerca di Roma 1, 00015 Monterotondo (RM), Italy; noemi.proietti@cnr.it (N.P.); valeria.ditullio@cnr.it (V.D.T.)
[5] KEMIA TAU SRL, Via Torino 56/64, 10040 La Cassa (Torino), Italy; rita.porcaro@kemiatau.com
[6] Department of Chemistry, Faculty of Sciences, University of Craiova, Calea Bucuresti Str. 107 I, 200512 Craiova, Romania
* Correspondence: elena.badea@edu.ucv.ro; Tel.: +40-734138920
† These authors contributed equally to this work.

Synthesis of OSA (oxidized sodium alginate)

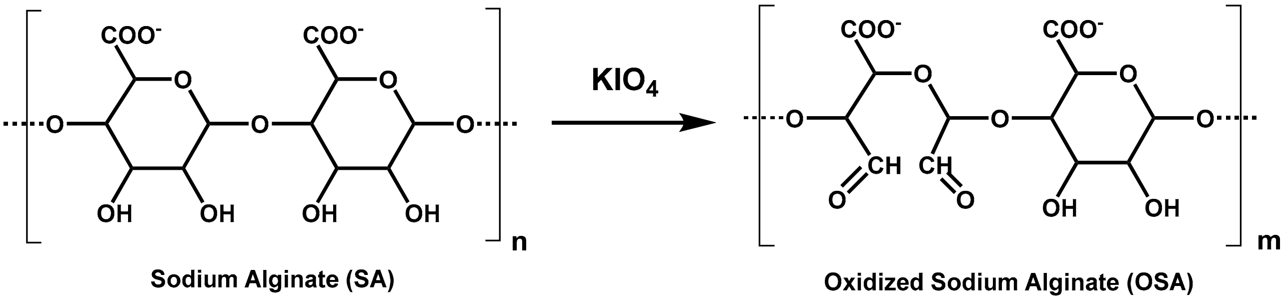

Figure S1. Oxidation of sodium alginate. Formation of aldehyde groups on main chain

Preparation method of nano-HAp at industrial level

The preparation method of nano-HAp at lab level was scaled up in Kemia Tau. Batches of 5 kg and 100 kg were prepared sequentially. The first step was the selection of the suitable reagents for industrial manufacturing. Since the use of ultrapure/pure reagents is possible only at laboratory scale, industrial alternatives were selected from Kemia Tau (Table S1).

Table S1. Reagents used for the preparation of nano-HAp at lab scale and at industrial scale (5 kg and 100 kg).

**Lab procedure (0.5 Kg)**	**Industrial scale up (5 -100 Kg)**
Calcium hydroxide [$Ca(OH)_2$] 96%	Hydrated lime
Phosphoric acid [H_3PO_4] 85%	Phosphoric acid 85%
Ammonium hydroxide 5N (NH_4OH)	Ammonia solution 33%
Distilled water	Mains water

For the 5 kg nano-HAp production, a big beaker and a propeller stirrer was used, in order to reproduce as close as possible the factory process (Figure S2A). For the scale up of 100 kg, the production was moved to the processing department, and cylindrical tanks equipped with propeller stirrers were employed. The final yield was more than 99%, with only a 0,73% of non-reacted hydrated lime (Figure S2B).

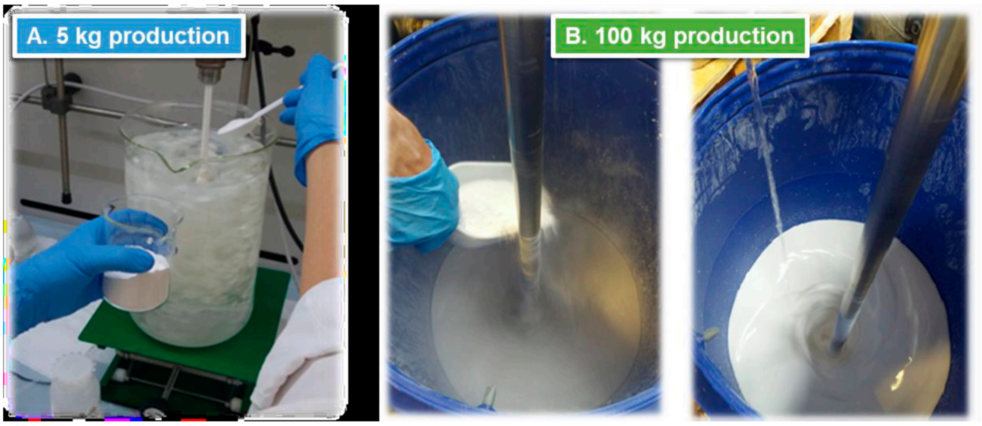

Figure S2. Nano-HAp production: A) 5 kg of nano-HAp produced using a big beaker and a propeller stirrer. B)100 kg of nano-HAp produced in the factory, using a cylindrical tank and a propeller stirrer. Both batches (5kg and 100kg) were analysed with the same characterization techniques used for the lab-scale samples, in order to assess the formation of HAp and its nano-dimensional size.

As the market require a shelf life of the products ≥12 M, different storage tests were performed to determine this parameter. The nano-HAp produced at industrial scale were stored in suspension and, after a few days, precipitation of nanoparticles was observed. This could be a problem for the potential formation of agglomerates and, most of all, for the applicative step. The use in leather processes of a not well-dispersed product requires a preliminary complex mixing phase that could affect the entire leather treatment.

In order to avoid the precipitation of nano-HAp particles, the viscosity of the suspension was increased adding different types of thickening agents (polymeric and/or inorganic agents). In order to find the best type and amount of thickening agents, a number of stability tests were performed. The products were subjected to accelerated aging tests, thermal stresses, and sunlight exposure tests.

The selected thickening agents gave a thixotropic effect to the formulation: under stationary conditions, the product has a viscosity such as to avoid separation; in dynamic conditions, the viscosity decreases making it easier to use.

The best results in accelerated aging tests was achieved with ACRYSOL TT -615 as thickening agent (12.4%), with a shelf life > 12M.

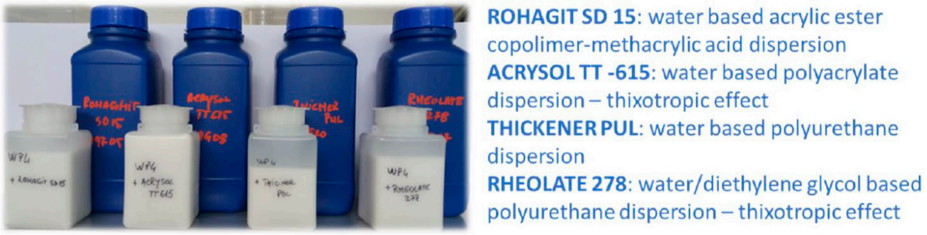

Figure S3. The selected thickening agents (ROHAGIT SD 15, ACRYSOL TT -615, THICKENER PUL, and RHEOLATE 278) added to the formulation.

Analysis on the nano-HAp: from laboratory to pilot-scale level

In Figure S4A, FTIR-ATR spectra of the nano-HAp produced at pilot scale are shown (blue and green spectra), compared to the spectrum of nano-HAp obtained at lab scale (black spectrum). In all cases typical signals of carbonates (1500-1400 cm^{-1}) and bulk phosphate groups are present (1090-960 cm^{-1}), showing only slight differences in the bands relative intensities.

Moreover, in Figure S4B, XRD patterns of the same samples are reported, showing the typical peaks of HAp single phase in all samples, with no significant differences in absolute/relative intensity and peak width. The absence of peaks related to extra phases is an indication of the purity of the HA material.

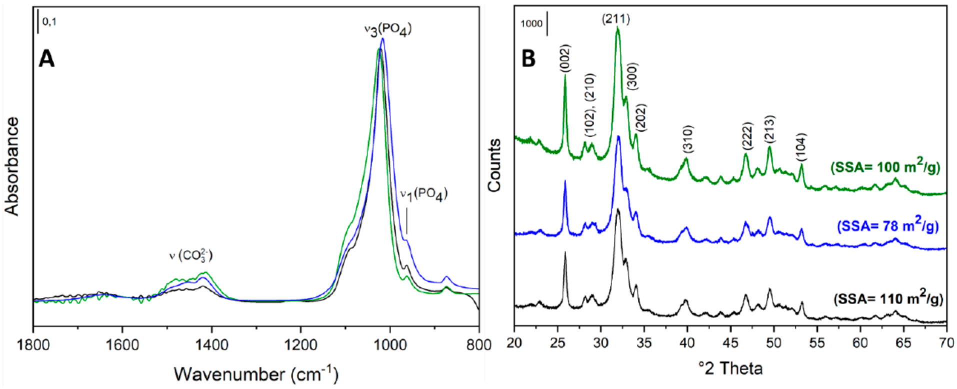

Figure S4. (A) FTIR-ATR spectra of nano-HAp at lab scale (black curve), nano-HpA scale up 5 kg (blue curve), and nano-HAp scale up 100 kg (green curve). (B): XRD patterns of nano-HAp at lab scale (black curve), nano-HAp scale up 5kg (blue curve), and nano-HAp scale up 100 kg (green curve). The specific surface area (SSA) values are reported above each curve.

In Figure S5, the shape and dimension observed by SEM, of the nano-HAp used in this study are reported.

It is possible to notice a tight agglomeration of the nanoparticles, and for a significant portion of the particles a needle-like shape can be appreciated, with size (length × width × thickness) of ca. 150-100 nm × 40-20 nm × 5-10 nm. The morphology of the nanoparticles was similar among different batches, confirming the reproducibility of the preparation. The nanometric size of HA observed by SEM was confirmed by specific surface area (SSA) values (between 110 and 115 m^2/g).

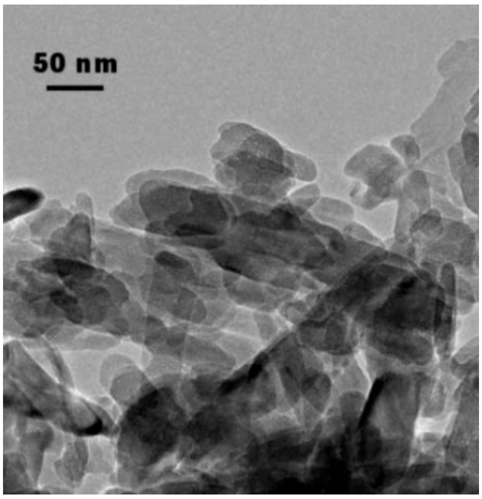

Figure S5. SEM image representative of the size and bulky morphology of the nano-HAp.

Laboratory tanning process using OSA and nano-HAp

A solution of OSA at molar ratio 0.8:1 was used by varying only the quantity of salt used. 10 g of un-pickled pelt was added, respectively, to a solution of 6% or 12% sodium chloride (calculated respect the weight of the hide) and 200 g of OSA solution and stirred for 2 hours at 25 °C at a pH between 5-6. Basification of the sample was realized by adding sodium bicarbonate, until the pH reached value 8. The samples were left to rest in the float for 18 hours before they were drained, rinsed and dried.

Table S2. Tanning process for testing the ability of OSA to bind to collagen (laboratory level)

Operation: Laboratory tanning process on calf leather[a,b]		
Products	% w/w	
Hide	100	T = 25 °C, pH= 5-6
NaCl	6 or 12	
OSA	2000	Rotate 2 hours
$NaHCO_3$	-	Rotate 30 min, pH = 8.0 ± 0.5 at 25°C
Leave in the tanning float 18 hours then drain, rinse and dry.		
[a] Prior to the tanning step, the calf hide was prepared following the classic preliminary physical-mechanical steps to obtain an un-pickled pelt to be used directly in the tanning process. [b] The percentage of added products is calculated based on the hide weight. That is, for 100 g hide, 100 g of water is added.		

The samples treaed with nano-HAp are obtained as follow: into 50 mL of solutions of 1, 1.5 and 3% nano-HAp in water, were added 5 g of leather tanned with OSA and stirred for 2 hours at 25 °C. Final pH value of the solution was around 9.3. The finals leathers were dried at room temperature.

NMR-mouse analysis of laboratory scale samples

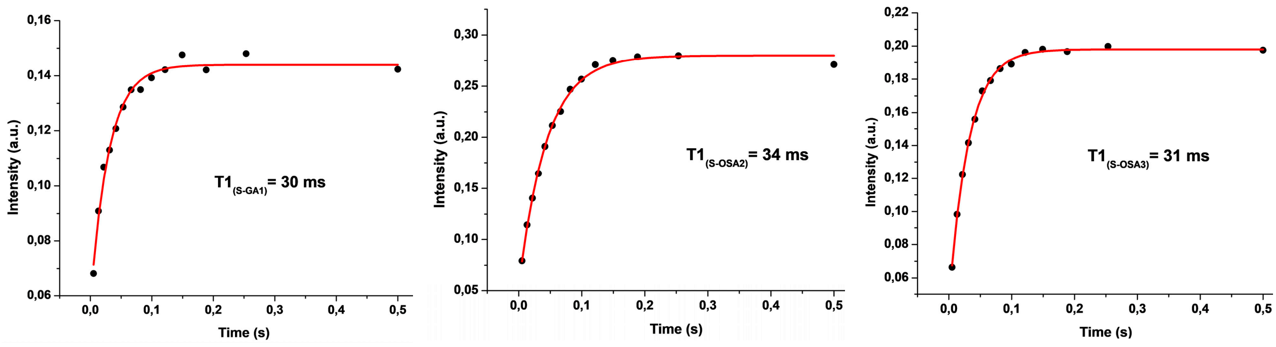

Figure S6. Longitudinal relaxation time (T_1) from CPMG signals of S-GA1, S-OSA2 and S-OSA3.

ATR-FTIR characterization of Oxidized sodium alginate (OSA) and Sodium alginate (SA)

Table S3. Attribution of the bands from the ATR-FTIR spectra of OSA and SA.

Wave number (cm^{-1})	Sodium alginate (SA)	Oxidized sodium alginate (OSA)
2853	Stretching vibrations of O–H bonds	Stretching vibrations of O–H bonds
1738	-	C=O stretching of aldehyde
1458	-	Symmetric stretching vibrations of carboxylate salt ion
1405	O–C–O symmetric stretching vibration	O–C–O symmetric stretching vibration
1141	-	Antisymmetric C-O-C mode
1024	C-O-C stretching vibration of pyranose	-
1015	-	C-O-C stretching vibration of pyranose
887	-	C_1–H deformation vibration of β-mannuronic acid residues
884	C_1–H deformation vibration of β-mannuronic acid residues	-
815	C-O-C of the alginate chains	-
786	-	Guluronic acid residues
716	-	Guluronic acid residues

Industrial scale-up of the tanning process using OSA and nano-HAp

Table S4. Tanning process with OSA at pilot-scale level

Operation: Pilot-scale tanning process on calf leather[a,b]		
Products	% w/w	
Hide	100	T = 25 °C, pH= 4.5-6.5
NaCl	6	
Stirring the hide with the NaCl for 20 minutes		
OSA	2000	Rotate 6-8 hours
Leave in the tanning float overnight		
$NaHCO_3$	-	Rotate 4-6 hours, pH = 8.0 ± 0.5 at 25°C
Leave in the tanning float 18 hours then drain, rinse and dry.		
[a] Prior to the tanning step, the calf hide was prepared following the classic preliminary physical-mechanical steps to obtain an un-pickled pelt to be used directly in the tanning process.<br>[b] The percentage of added products is calculated based on the hide weight. That is, for 100 g hide, 100 g of water is added.		

The suspension of nano-HAp was applied using the roll coater technology. The roller finishing machine or *Roll Coater* consists of an endless rubber roller that brings the leather into contact with a metal cylinder engraved to a certain texture: the finishing liquid is stretched and smeared by pressure on the leather through direct contact with the cylinder, called "gravure cylinder" (Figure S7A). The cylinder is wetted with the finishing solution contained in the mix tank and, after scraping the excess liquid, compresses the leather by depositing the finishing layer on it. The engraving of the cylinder consists of several alveoli that contain the finishing liquid and the amount deposited on the leather depends on the size, number of alveoli per unit surface and the rate of rotation of the gravure cylinder. This allows a uniform distribution over the entire surface of the leather and avoids waste of a finishing solution that remains deposited on the gravure and recovered cylinder. A rubber transport cylinder (support roller) ensures the perfect contact between the leather and the cylinder. In this specific case, the "Reverse" application technique in which the gravure cylinder rotates in the opposite direction to that of the leather rubber belt was used (Figure S7B).

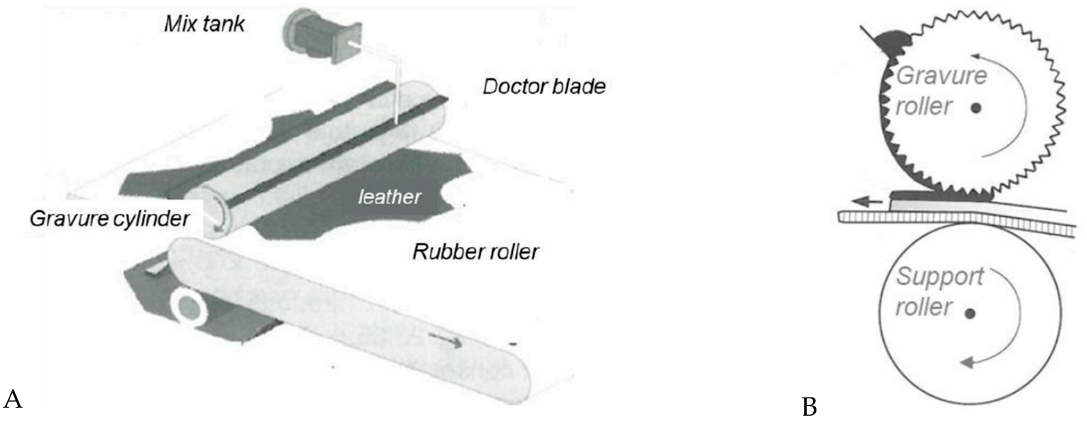

Figure S7. (A) Representation scheme of the Roll Coater (B) On the left: scheme of the rotation of the gravure roller and the support roller in the "reverse" application. (images provided by Kemia Tau).

NMR-mouse analysis of industrial scale samples

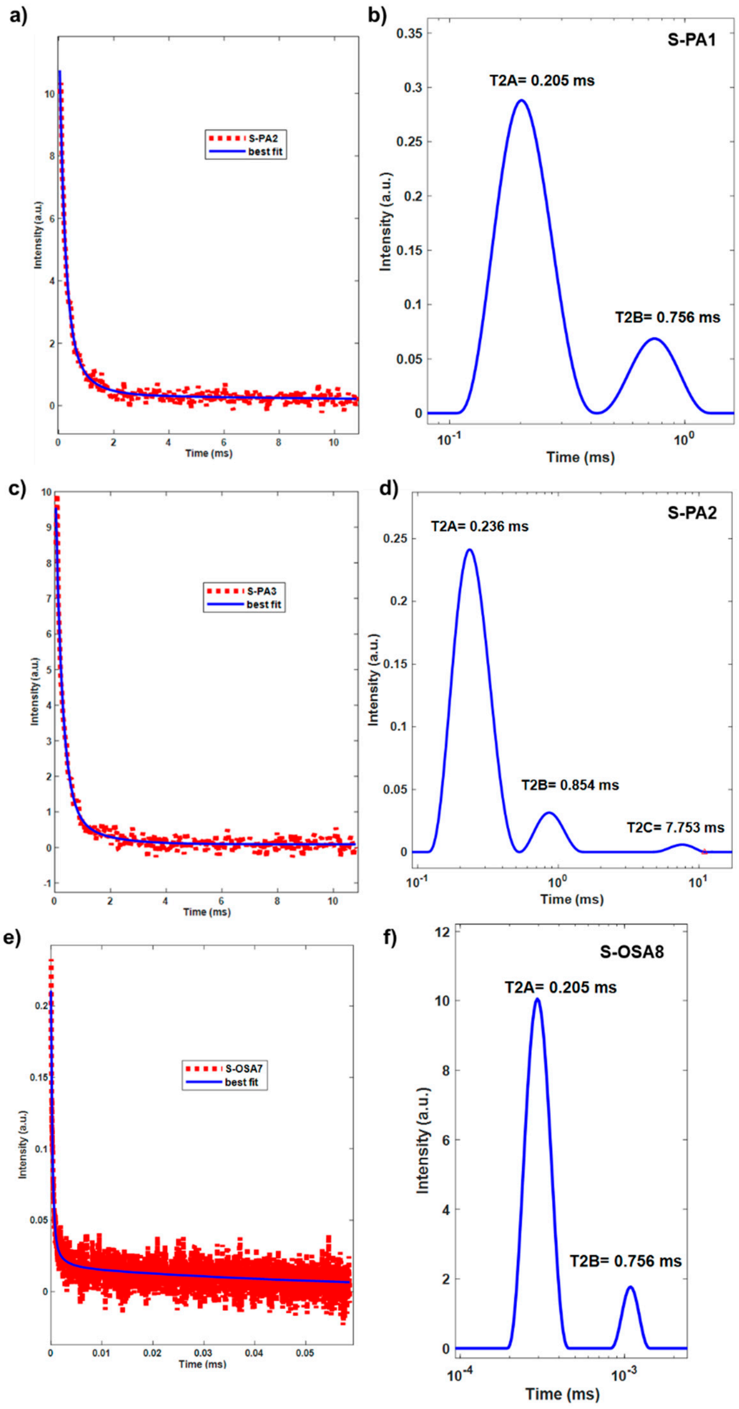

Figure S8. Longitudinal relaxation time (T_1) from CPMG signals of (a) S-PA1, (c) S-PA2, (e) S-OSA7 and transverse relaxation time (T_2) distribution for the CPMG signals calculated with inverse Laplace of (b) S-PA1, (d) S-PA2, (f) S-OSA7.

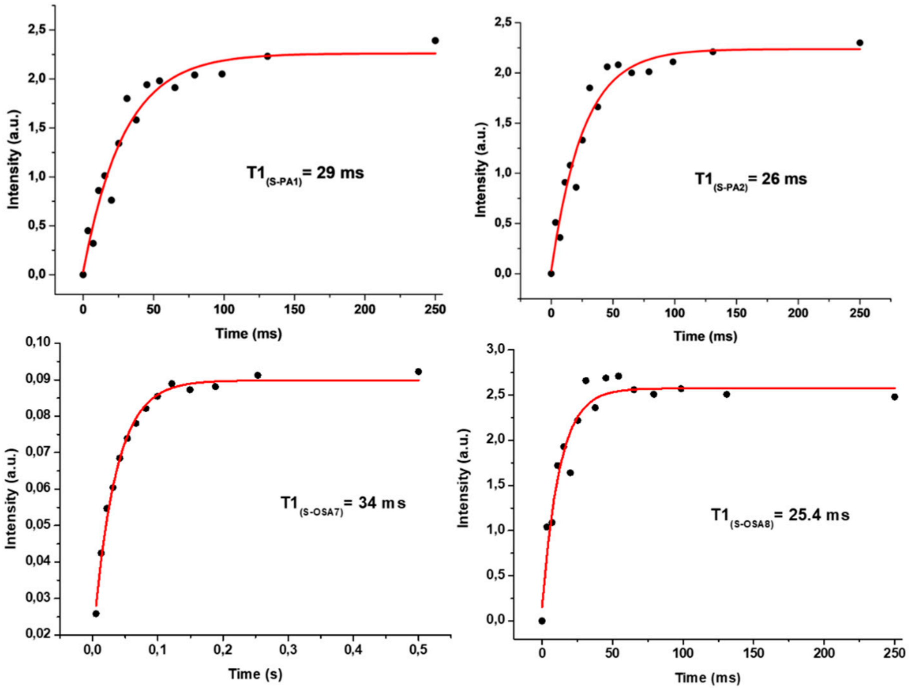

Figure S9. Longitudinal relaxation time (T1) from CPMG signals of S-PA1,S-PA2 S-OSA7 and S-OSA8.
